# Minimally Invasive Transforaminal Lumbar Interbody Fusion and Unilateral Fixation for Degenerative Lumbar Disease

**DOI:** 10.1111/os.12345

**Published:** 2017-09-27

**Authors:** Hui‐wang Wang, Yong‐cheng Hu, Zhan‐yong Wu, Hua‐rong Wu, Chun‐fu Wu, Lian‐suo Zhang, Wei‐kun Xu, Hui‐long Fan, Jin‐sheng Cai, Jian‐qing Ma

**Affiliations:** ^1^ Department of Orthopaedics Orthopaedic Hospital of Xingtai Xingtai China; ^2^ Department of Spinal Surgery Tianjin Hospital Tianjin China; ^3^ Department of Orthopedic Laboratory Xingtai Institute of Orthopaedics Xingtai China

**Keywords:** Fusion rate, Lumbar degenerative disease, Lumbar interbody fusion, Posterolateral fusion, Unilateral pedicle screw fixation

## Abstract

**Objective:**

To evaluate the clinical effect of the minimally invasive transforaminal lumbar interbody fusion combined with posterolateral fusion and unilateral fixation using a tubular retractor in the management of degenerative lumbar disease.

**Methods:**

A retrospective analysis was conducted to analyze the clinical outcome of 58 degenerative lumbar disease patients who were treated with minimally invasive transforaminal lumbar interbody fusion combined with posterolateral fusion and unilateral fixation during December 2012 to January 2015. The spine was unilaterally approached through a 3.0‐cm skin incision centered on the disc space, located 2.5 cm lateral to the midline, and the multifidus muscles and longissimus dorsi were stripped off. After transforaminal lumbar interbody fusion and posterolateral fusion the unilateral pedicle screw fixation was performed. The visual analogue scale (VAS) for back and leg pain, the Oswestry disability index (ODI), and the MacNab score were applied to evaluate clinical effects. The operation time, peri‐operative bleeding, postoperative time in bed, hospitalization costs, and the change in the intervertebral height were analyzed. Radiological fusion based on the Bridwell grading system was also assessed at the last follow‐up. The quality of life of the patients before and after the operation was assessed using the short form‐36 scale (SF‐36).

**Results:**

Fifty‐eight operations were successfully performed, and no nerve root injury or dural tear occurred. The average operation time was 138 ± 33 min, intraoperative blood loss was 126 ± 50 mL, the duration from surgery to getting out of bed was 46 ± 8 h, and hospitalization cost was 1.6 ± 0.2 ten thousand yuan. All of the 58 patients were followed up for 7–31 months, with an average of 14.6 months. The postoperative VAS scores and ODI score were significantly improved compared with preoperative data (P < 0.05). The evaluation of the MacNab score was excellent in 41 patients, good in 15, and fair in 2, suggesting an effective rate of 96.6%. The intervertebral height had reduced 0.2 ± 1.2 mm by the last follow‐up, and there were 55 Grade I and II cases based on the Bridwell evaluation criterion. The fusion rate was 94.8%, and no screw breakage and loosening occurred. The scores of physical pain, general health, social, and emotional functioning were significantly increased at the last follow‐up.

**Conclusion:**

Minimally invasive transforaminal lumbar interbody fusion combined with posterolateral fusion and unilateral fixation provide a new choice for degenerative lumbar disease, and the short‐term clinical outcome is satisfactory.

## Introduction

Symptomatic lumbar degenerative disease is essentially characterized by pains and walking difficulties due to abnormal motion or compression of neural structures and their vessels. This reflects specific situations, such as narrowing of the spinal canal, degenerative disc disease, and herniated discs, as well as any degenerative impairment of the posterior arch (e.g. arthropathy and spondylolisthesis). Most of the time surgical treatment is necessary to reduce the symptoms, with arthrodesis generally regarded as being the treatment of choice for this pathology^1^, ^2^.

Kabin first reported the unilateral fixation in 1990 at The North American Spine Society (NASS), and the unilateral fixation was used in clinic in 1992. Thirty‐six patients were retrospectively followed for an average of 25.1 months to evaluate the relative effectiveness of unilateral (16 patients) versus bilateral (20 patients) variable screw placement (VSP) instrumentation in isolate L_4_–L_5_ fusions. Clinical outcome, as obtained through standardized measurement techniques of pain and function, demonstrated 69% excellent and good results. Fusion results with unilateral instrumentation were nearly identical to those of bilateral^3^. Fernández‐Fairen *et al*. also conducted a prospective randomized comparative clinical study^4^. The degenerative lumbar spondylolisthesis patients were treated with posterolateral fusion (PLF) and followed up for 3 years. There was no internal fixation failure, the operation time was significantly shorter in the unilateral fixed than in the bilateral fixed treatment, and the rate of good clinical efficacy, the bone graft fusion rate, and the adjacent segment degeneration were not statistically significantly different between the two groups^4^. The clinical effect of unilateral pedicle screw with single cage interbody fusion in minimally invasive posterior lumbar interbody fusion (PLIF) , was similar to the bilateral pedicle screw with double cage fixation, but the operation time, blood loss, and cost were less than the bilateral fixed treatment^5^.

The minimally transforaminal lumbar interbody fusion (Mis‐TLIF) was first reported by Foley and Lefkowitz in 2002; in recent years, it has been widely used, with satisfactory outcomes obtained^6^, ^7^. In this surgery, the working channel was built by expanding the tubular retractor to gradually extend the channel. The decompressing, intervertebral fusion and internal fixation were performed under direct vision, so this method has some advantages, including high safety and minimal trauma, and reduced muscular dissection results in smaller wounds, less tissue trauma, and faster recovery^8^, ^9^. This technology usually involves unilateral or bilateral spinal canal decompression combined with bilateral fixation; however, there are few reports on unilateral fixation^10^.

Few studies have focused on the unilateral minimally invasive transforaminal lumbar interbody fusion, and the overall aim of the present study was to evaluate the clinical effect of the minimally invasive transforaminal lumbar interbody fusion combined with posterolateral fusion and unilateral fixation using a tubular retractor in the management of degenerative lumbar disease.

## Methods

### *Inclusion and Exclusion Criteria*

Inclusion criteria included: (i) typical symptom of low back pain and lower limb pain; (ii) radiological examination to support diagnosis; (iii) single segment lesion or committed segment was single segment level; (iv) conservative treatment had failed after more than 6 months; (v) extreme lateral lumbar disc herniation or large herniated disk along with or without instability of lumbar; and (vi) lumbar spinal stenosis with unilateral symptoms.

Exclusion criteria: (i) lumbar spondylolisthesis and (ii) lumbar infection, tumor, congenital lumbar spinal stenosis, serious osteoporosis and malformation.

### *Patients’ Information*

A total of 67 patients who underwent minimally invasive transforaminal lumbar interbody and unilateral fixation at the Orthopedic Hospital of Xingtai between December 2012 and January 2015 were retrospectively reviewed. Fifty‐eight patients met the entry criterion; there were 32 men and 26 women, with an average age of 62 years (range, 51–74 years), including 43 cases of lumbar disc herniation (LDH) (with 2 cases of extreme lateral lumbar disc herniation) and 15 cases of lumbar spinal stenosis. Among them, the level of herniation was L_3–4_ in 13 cases, L_4–5_ in 27 cases, and L_5_–S_1_ in 18 cases. All of the cases were single segment level lesions, including 46 cases with unilateral lower limbs symptoms and 12 cases with bilateral lower limb symptoms.

### *Surgical Methods*

**Figure 1:**
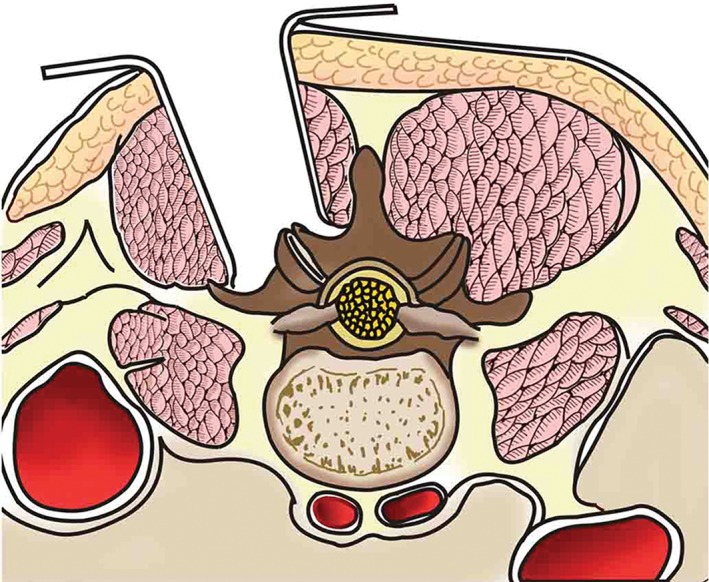
The cross‐section diagram of lumbar applying outspread channel. The multifidus muscles and longissimus dorsi were stripped off. After making an approach between those muscles, a tubular retractor was placed on the lamina and the facet joint.

**Figure 2:**
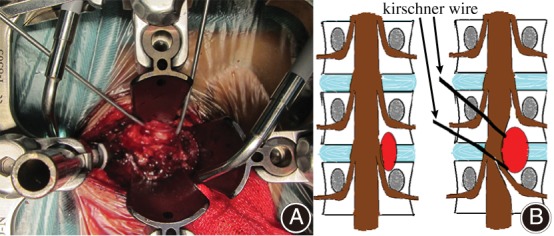
Intraoperative images of the working channel. (A) The inferior articular facet and the upper half of the superior articular facet were removed. (B) Two Kirschner wires were used to drag the nerve root to the middle, to prevent nerve injury during the decompression.

Patients were placed in the prone position on a radiolucent table after anesthesia. The pedicles of the vertebral arch of the operated level were marked on the skin with a C‐arm machine. A 3.0‐cm skin incision was made, which was located 2.5 cm lateral to the midline. The lumbar fascia was then incised, and the finger fracture technique was used to dissociate the muscle fibers until contact was made with the facet joint. A retractor was placed after progressively larger dilating bougies were placed. The inferior and superior articular processes and part of the vertebral lamina, as well as part of the base of the spinous process, were removed with a high‐speed drill or osteotome. These bones were kept for use as an autograft during interbody fusion. The nerve root was decompressed by removal of the ligamentum flavum and bone spur. A sharp knife was used to create a window on the annulus fibrosus. Exeresis of the disc was performed until contact was made with the anterior longitudinal ligament, and progressive intervertebral distraction was performed using progressively larger dilating bougies. The local autograft was implanted after one cage implant, and two pedicle screws were inserted and fixed on the ipsilateral side (Figs 1, 2).

**Figure 3:**
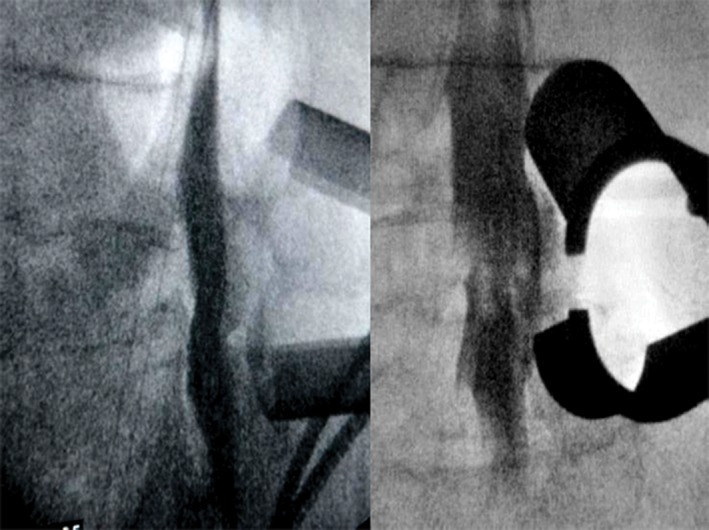
The patient (57 years old, female) had bilateral lower limb symptoms, which were treated with unilateral incision stealth bilateral intraoperative spinal canal decompression, and the myelography showed that the dural sac and nerve root contrast filling was good, without obvious press signs. It was not necessary to perform lateral incision decompression.

**Figure 4:**
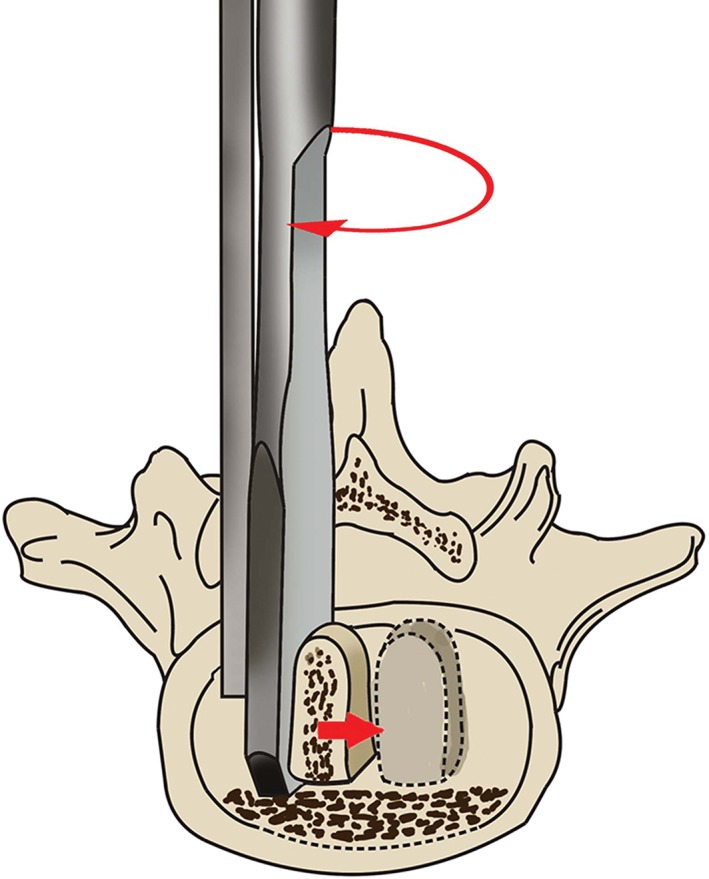
Diagram of bone grafting. The bone grafting funnel was used to graft autogenous spongy bone and then the cortical iliac blocks were implanted.

**Figure 5:**
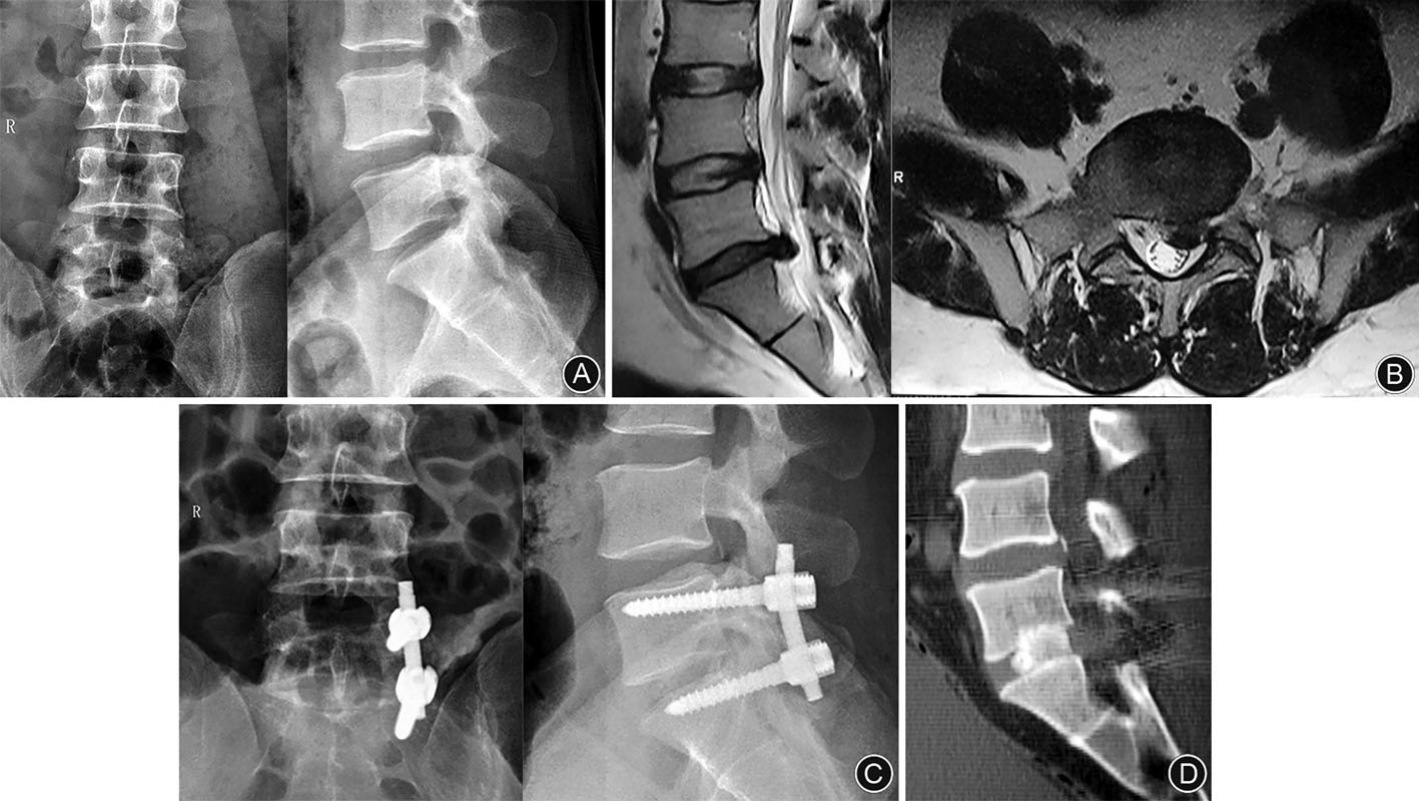
Clinical imaging from one representative patient (male, 56 years old) who complained of lumbago and left lower limb extremity pain for approximately 6 months. (A) X‐ray images of lumbar vertebrae anteroposterior and lateral position plain films showed no vertebral olisthy. (B) T2‐weighted sagittal (left) and axial (right) preoperative magnetic resonance images (MRI) of the lumbar spine showed the L_5_–S_1_ disc herniation. (C) The X‐ray imaging indicated that the location of the internal fixator was excellent. (D) The lumbar vertebrae CT indicates that the location of the grafting bone block with intervertebral space was fine, and the synostosis of intervertebral space was also excellent.

A myelography was used to estimate the intervertebral space following the above operative steps. The operator continued to clean the intervertebral space if the area was not cleaned completely, with the purpose to accelerate the fusion rate (Fig. 3). If the contralateral was compressed, the spinous process root was cut off, to reveal and cut off ligamentum flavum, and then the contralateral lateral recess was decompressed. After the foramen intervertebrale was decompressed thoroughly, using the bone grafting funnel to graft part of trivial osseous blocks and cortical iliac blocks which three sides were repaired (Fig. 4). Finally, we explored and checked the relaxed nerve root, used the absorbable gelatin sponge for the hemostasis, took out the retractor, and imbedded the pedicle screw, and the C‐arm was used to confirm the entry point of the lower centrum’s pedicle screw. The reflexed waist protrusion pitman was installed and pressed moderately to recovery the lumbar lordosis and prevent the bone graft from moving. The bed was made for grafting bone. A drain was placed to prevent epidural hematoma after surgery and then the incision was sutured (Fig. 5).

### *Postoperative Treatment*

The patients were treated by antibiotic therapy for 24 h following surgery. One day post‐operation, the patient started to ambulate with the protection of a girdle. The brace continued to be worn for approximately 3 months, and aggravating activities and carrying weight were to be avoided.

### *Follow‐up Method and Therapeutic Evaluation*

The retrospective inspection was carried out after 3 and 6 months post‐operation, and the last follow‐up visit (informed by calling). The visual analogue scale (VAS) method, the Oswestry disability index (ODI) index, and the Macnab scoring system were used to evaluate the clinical effects during post‐operative check‐ups at 3 days, 3 months, 6 months, and the last follow‐up visit.

The status of intervertebral fusion was measured using the Bidwell evaluation criterion at final follow up. The evaluation was made as follows: Grade I, fused with remodeling and trabeculae; Grade II, graft intact, not fully remodeled and incorporated though but with no lucencies above or below; Grade III, graft intact but a definite lucency at the top or bottom of the graft; and Grade IV, definitely not fused with resorption of bone graft and collapse^11^.

### *Statistical Analysis*

Statistical analysis on all parameters was performed using SPSS13.0 software (SPSS, Chicago, IL, USA). Data were expressed as mean and standard deviation (mean ± SD) and were compared using the paired samples *t*‐test. A *P*‐value less than 0.05 was considered statistically significant.

## Result

### *General Condition and Clinical Outcomes*

The operative time for the patients was 138 ± 33 min (range, 97–185 min), with an average intraoperative blood loss of 126 ± 50 mL (range, 65–210 mL). The time that patients needed to get out of bed after surgery was 46 ± 8 h (range, 38–76 h), and the hospitalization expense was 1.6 ± 0.2 ten thousand yuan (range, 1.2–2.1 ten thousand yuan). All 58 patients showed up at the scheduled follow‐up visits, and the follow‐up period was 7–31 months (mean, 14.6 months). The SF‐36 scale of 58 patients confirmed that the scores for physical pain, general health, and social and emotional functioning had improved obviously by the last follow‐up. Compared with before surgery, physical pain decreased by 43.6%, general health increased 46.4%, social functioning increased 40.1%, and emotional functioning increased 48.7%. However, there was no significant difference preoperatively and postoperatively in regards to physiological function, physical role, vitality, and the mental health score (Table 1).

**Table 1:** The SF‐36 score for 58 cases at preoperation and last follow‐up ($\bar{x}\pm s$)

Time	Physiological function	Physical role	Physical pain	General health	Vitality	Social functioning	Emotional functioning	Mental health
Preoperation	58.5 ± 5.9	47.5 ± 2.8	47.3 ± 3.4	46.3 ± 4.5	54.5 ± 5.4	50.4 ± 2.4	46.6 ± 2.7	64.5 ± 7.3
Post‐operation	69.3 ± 6.7	48.0 ± 3.8	67.9 ± 4.7	67.8 ± 4.3	55.2 ± 5.9	70.6 ± 4.8	69.3 ± 3.7	65.4 ± 6.7
*t‐value*	−1.34	−1.56	−35.53	−32.35	−0.63	−30.30	−49.13	−1.15
*P‐value*	0.187	0.124	0.000	0.000	0.532	0.000	0.000	0.256

### *Therapeutic Evaluation*

The VAS scores for back pain were 2.3 ± 1.3 3 days post‐operation, 1.3 ± 0.8 3 months post‐operation, 1.8 ± 0.7 6 months post‐operation, and 1.2 ± 0.7 at the last follow‐up, all of which were significantly lower than that recorded preoperatively (4.6 ± 1.0; *P* < 0.05) (Table 2). Compared with preoperative data, the back pain VAS score at 3 days post‐operation decreased 50%, at 3 months decreased 71.7%, at 6 months decreased 60.9%, and at last follow‐up decreased 73.9%.

**Table 2:** Preoperative and postoperative visual analogue scale (VAS) and Oswestry disability index (ODI) scores ($\bar{x}\pm s$)

Methods	Preoperation	After 3 days	After 3 months	After 6 months	At last follow‐up
VAS (back)	4.6 ± 1.0	2.3 ± 1.3	1.3 ± 0.8	1.8 ± 0.7	1.2 ± 0.7
*t‐*value		19.821	26.733	22.115	29.32
*P‐*value		0.000*	0.000*	0.000*	0.000*
VAS (leg)	6.8 ± 1.3	1.9 ± 1.2	2.0 ± 1.1	1.6 ± 1.3	1.9 ± 1.2
*t‐*value		38.438	38.456	41.944	32.559
*P*‐value		0.000*	0.000*	0.000*	0.000*
ODI index	38.6 ± 6.3	16.5 ± 4.3	15.6 ± 5.1	16.2 ± 5.4	14.8 ± 5.3
*t‐*value		23.892	22.673	20.968	22.583
*P‐*value		0.000*	0.000*	0.000*	0.000*

* Compared with preoperative data.

The VAS scores for lower limb pain were 1.9 ± 1.2 3 days post‐operation, 2.0 ± 1.1 3 months post‐operation, 1.6 ± 1.3 6 months post‐operation, and 1.9 ± 1.2 at the last follow‐up visit, all of which were also significantly lower than that recorded preoperatively (6.8 ± 1.3; *P* < 0.05) (Table 2). Compared with preoperative data, the lower limb pain VAS score at day 3 post‐operation had decreased 72.1%, by 3 months had decreased 80%, by 6 months had decreased 76.5%, and at last follow‐up had decreased 72.1%.

The ODI was 16.5 ± 4.3 3 days post‐operation, 15.6 ± 5.1 3 months post‐operation, 16.2 ± 5.4 6 months post‐operation, and 14.8 ± 5.3 on the last follow‐up visit, all of which were also significantly lower than that recorded preoperatively (38.6 ± 6.3; *P* < 0.05) (Table 2). Compared with preoperative data, the ODI score 3 days post‐operation had decreased by 57.3%, by 3 months had decreased by 59.6%, at 6 months had decreased by 58.3%, and at last follow‐up had decreased by 61.7%.

Following the Macnab standard of evaluation, 41 patients’ results were excellent, fine for 2 patients, and were tolerable for 2 patients. The satisfaction rate was 96.6%.

### *Intervertebral Height and Fusion*

The measuring method of intervertebral height was to measure the anterior and posterior edge of intervertebral space using standard lateral X‐ray imaging; the intervertebral height was the mean value of these. The intervertebral height was 6.4 ± 1.6 mm 3 days post‐operation, 6.2 ± 1.4 mm 3 months post‐operation, 6.1 ± 1.2 mm 6 months post‐operation, and 6.2 ± 1.3 mm on the last follow‐up visit, all of which were similar preoperation (*P* > 0.05). The intervertebral height of the last follow‐up visit fell 0.2 ± 1.2 mm compared with 3 days post‐operation, but there was no statistical significance (*P* > 0.05).

According to the Bridwell evaluation criterion of intervertebral fusion, there were 38 cases of Grade I (65.6%), 17 cases of Grade II (29.3%), and 3 of Grade Ш (5.2%); the fusion rate was 94.8%. No screw breakage or loosening occurred. The three cases whose evaluation criterion were Grade Ш had no other clinical symptoms, and they were still followed up.

### *Complications*

Among 58 cases, there were 2 cases of skin flay necrosis; these were found 3 days after surgery and were resolved by dressing change. Eight cases had bone took zone pain around the iliac crest, with the symptom relieved after 3 months of treatment with non‐steroid anti‐inflammatory drugs.

## Discussion

Among various spinal fusion techniques, TLIF has become a popular and established technique because it can reduce the amount of thecal sac and nerve root retraction through the lateral approach to the disc space^12^, ^13^, ^14^. Although the open TLIF procedure preserves the major portion of the posterior ligament complex with minimal compromise of spinal stability, it also requires dissecting and retracting paraspinal muscle, which can cause muscle denervation, atrophy and, consequently, postoperative low back pain^15^. With the advent of modern image guidance and sophisticated instrumentation, the MIS‐TLIF was introduced by Foley and Lefkowitz for the first time in 2002^6^. Since then, it has become an increasingly popular technique and has been proven advantageous to traditional open surgery in terms of damage upon spinal soft tissues^14^, ^16^.

Numerous previous biomechanical studies have attempted to comparatively evaluate the unilateral and bilateral pedicle screws (PS) fixation approach, and inconsistent results have been obtained. Chen *et al.* demonstrated that unilateral pedicle screws (UPS) fixation was adequate to maintain the stability of the spine in a biomechanics study^17^. On one hand, similar studies confirmed that the UPS system was effective to reduce stress shielding of the vertebra and diminish peak stress arising in the adjacent levels above and below the fusion, and UPS fixation resulted with a lower incidence of adjacent‐segment degeneration than bilateral pedicle screws (BPS) fixation^18^. We also performed some biomechanical testing to determine whether the two fixed methods could attain the same mechanical stability in treating lumbar degenerative disease^19^, ^20^. However, on the other hand, Aoki *et al.* observed that UPS fixation caused postoperative cage migration more frequently than BPS fixation^21^. Another study found that UPS fixation supplied only half of the improvement in stiffness compared with BPS fixation and caused significant off axis rotational motions, which could be detrimental to stability and fusion after TLIF^22^. Similar to the biomechanical research, the conclusions of clinical studies are controversial. A prospective study of 87 patients demonstrated that the UPS fixation was as effective as BPS fixation in lumbar spinal fusion independent of the number of fusion segments (one or two segments) or pedicle screw systems^23^. Another study reported that UPS instrumented TLIF is a safe, feasible, and viable treatment option generating better results, especially in terms of operative time, blood loss, and hospital time for single‐level disease and implant costs. No decrease in the fusion rate or increase in the complication rate was observed during 2 years of follow‐up^10^, ^24^. In our study, we used unilateral fixation for degenerative lumbar disease, and the short clinical outcome is satisfactory. Unilateral pedicle screws fixation proved adequate to maintain the stability for the fusion.

In our study, 58 patients were treated with minimally invasive transforaminal lumbar interbody fusion and unilaterally fixed with polyaxial screws. We found that the polyaxial screws were stronger than the monoaxial pedicle screws. Half or two‐thirds of the inferior articular facet was removed during the decompression, and part of the inferior articular facet was retained, which could help to maintain the spinal stability. We used the method of combining interbody fusion with posterolateral grafting to enhance the spinal stability.

For the patients with bilateral neurologic symptoms, we adopted the method that was described by Gu *et al.*, which involves cutting off the spinous process root and performing extensive decompression of the contralateral side, including the central stenosis, the ligamentum flavum and its bony attachment, the deep cortical surface of the contralateral lamina, and the contralateral lateral recess and foramen^25^. Although the minimally invasive transforaminal lumbar interbody fusion has some advantages, it was still limited. The major issue was too little room to work in the channel. Decompression, bone grafting, and internal fixation were difficult to carry out in the limited space. To avoid the above problems, we designed a new method of traction and pulling the dural sac and nerve root with the help of Kirschner wires, which were fixed on the centrum. This method protects the dural sac and nerve root from injury, and the operator could work with two hands, thus reducing the number of operators and operative time. For the purpose of improving the safety of the surgery, electromyography monitoring was used to guarantee that there were no complications after surgery (e.g. iatrogenic nerve root injury; Fig. 2). To avoid the incision skin was persistently pressed, which may caused skin flay necrosis, we suggest that the tubular retractor should be loosened about 5 min if the operation time was too long.

Based on the clinical follow‐up research, our opinion is that the minimally invasive transforaminal lumbar interbody fusion combined with posterolateral fusion and unilateral fixation (Mis‐TLIF + PLF) for the treatment of lumbar degenerative disease has some advantages, including minimal trauma, quicker postoperative recovery, being simple to master for the beginner, and satisfactory curative effect. The approach obtained satisfactory clinical effects in the short term; however, persistent follow‐up is needed to investigate long‐term outcomes.
